# Response to Letter to the Editor: “Re-evaluating the link between internet use during pregnancy and low birth weight in light of maternal mental health”

**DOI:** 10.1265/ehpm.25-00238

**Published:** 2025-08-06

**Authors:** Aya Sakakihara, Chiyori Haga, Aya Kinjo, Yoneatsu Osaki

**Affiliations:** 1 Department of Community Health Nursing, Faculty of Medicine, Shimane University, 89-1 Enya-cho, Izumo city, Shimane Prefecture 693-8501, Japan; 2 Department of Community Nursing, Graduate School of Medicine, Kagawa University, 1750-1 Ikenobe, Miki-cho, Kita-gun, Kagawa Prefecture 761-0793, Japan; 3 Division of Environmental and Preventive Medicine, Faculty of Medicine, Tottori University, 86 Nishi-cho, Yonago city, Tottori Prefecture 683-8503, Japan

To the Editors,

Nagahide Takahashi, Akemi Okumura, Chika Kubota, and Kenji J. Tsuchiya,

We are sincerely grateful for your interest in our recent publication, “Association between long Internet use during pregnancy and low birth weight: a retrospective cohort study” [1].

We appreciate your thoughtful commentary [2] on the potential mediating and confounding roles of maternal mental health in the observed association between prolonged internet use during pregnancy and low birth weight (LBW), as well as your suggestions concerning the mechanisms underlying this association.

As indicated, internet usage has become an indispensable part of contemporary daily life. We acknowledge that the results of our study do not provide grounds to advocate for the restriction of internet use during pregnancy. Rather than simply viewing prolonged Internet use as problematic behavior, we believe it is important to consider the underlying factors that may lead pregnant women to such use and to regard this understanding as a potential entry point for providing thoughtful and appropriate support to those in need.

Our investigation utilized existing data obtained at the times of pregnancy and birth registration in Matsue city, Shimane Prefecture, Japan. These data are not intended for clinical screening, but instead are used by public health nurses and other community-based healthcare professionals to identify individuals potentially in need of early interventions. Such data are designed are designed for primary and secondary prevention efforts that support pregnant women who are in need of assistance and connect them with appropriate medical services when necessary. Consequently, our dataset did not include information on psychiatric or developmental disorders or genetic predispositions.

Therefore, we are unable to further examine the mechanisms underlying the association between internet use and LBW, particularly with regards to maternal psychopathology. We agree that this represents a meaningful direction for future research.

With full awareness of these limitations, we would still like to emphasize that the content of our study is internally consistent and carefully considered. In response to your observations, we would like to provide clarification on the rationale underlying our interpretations.

The reason we did not describe the inability to include maternal mental disorders as covariates as a limitation of this study, nor did we address maternal mental health in our discussion of the mechanisms underlying the association between prolonged internet use during pregnancy and LBW, is that these relationships remain under debate and have not yet been clearly established.

For example, beyond the indicated study by Murray et al. [3], there is limited literature on the association between maternal ADHD and LBW. While it has been suggested that ADHD may affect prenatal care adherence, Ninowski et al. [4] reported no association between ADHD and adverse maternal behaviors, such as a poor diet, smoking, or alcohol consumption.

In addition, regarding the association between maternal depression or anxiety symptoms during pregnancy and the incidence of LBW, a systematic review and meta-analysis by Grigoriadis et al. [5] reported no significant association between maternal depression and LBW. In addition, studies from several high-income countries, including the United States, Sweden, Norway, and Germany, have reported no significant association between maternal depression or anxiety symptoms during pregnancy and the incidence of LBW [6–9].

Furthermore, according to a systematic review and meta-analysis of sleep disturbances during pregnancy and their associations with adverse maternal and fetal outcomes, sleep disturbances were associated with conditions such as gestational hypertension, gestational diabetes, and large for gestational age, but not with small for gestational age or LBW [10]. On the other hand, extensive evidence has been accumulated on the relationship between poor maternal nutrition or inadequate gestational weight gain and LBW [11]. In Japan, maternal nutrition, dietary quality, and the status of prenatal care attendance have been identified as major factors contributing to LBW [12–15]. Therefore, we adopted these factors as potential mechanisms underlying prolonged Internet use during pregnancy. Based on these considerations, our discussion was carefully developed, drawing solely on the most robust evidence.

However, as indicated, we cannot rule out the potential of maternal psychopathology as a confounding factor in the association between prolonged Internet use during pregnancy and LBW. Therefore, we consider it important to take maternal psychopathology into account in future studies that aim to examine this relationship in more detail.

The benefits of Internet use are considerable, and for pregnant women as well, it serves as a valuable tool for gathering information, communication, and relaxation. In the present study, we were limited to analyzing Internet usage times only based on existing data. However, as suggested, we recognize the importance of investigating the psychological background underlying Internet use in future research. This will help us to obtain a more detailed understanding of the characteristics and circumstances of those who may require support among pregnant women with prolonged Internet use. Furthermore, such an approach will help avoid stigmatizing Internet use itself and instead promote thoughtful consideration of how pregnant women may engage with the Internet in a healthy and constructive manner.

The only conclusion that may be drawn from the present study is that prolonged Internet use during pregnancy was associated with LBW. It remains unclear whether this reflects a causal relationship. Nonetheless, focusing on prolonged Internet use during pregnancy may serve as a useful indicator for identifying individuals who may require support, including the possibility of underlying mental health issues. Given that reducing the incidence of LBW remains an important public health goal, such an approach could serve as a meaningful reference for early intervention efforts. This study was driven by the desire to generate evidence that may be practically applied by public health nurses, midwives, and other maternal care professionals, in order to facilitate early interventions.

As pointed out in your letter, the idea of viewing prolonged Internet use during pregnancy as a potential clinical signal is also addressed in our original paper. We believe it is essential to interpret this behavior in the context of a woman’s broader living circumstances and mental health status, and to provide the necessary support accordingly.

We will strive to conduct studies designed to address the limitations of the present research and to provide more specific clinical and policy recommendations for pregnant women.

Once again, we express our sincere appreciation for your insightful and constructive feedback, which has provided valuable direction for future investigations in this field.


Sincerely, Aya Sakakihara, Chiyori Haga, Aya Kinjo, Yoneatsu Osaki

